# Vitamin B6-Dependent Enzymes in the Human Malaria Parasite *Plasmodium falciparum*: A Druggable Target?

**DOI:** 10.1155/2014/108516

**Published:** 2014-01-09

**Authors:** Thales Kronenberger, Jasmin Lindner, Kamila A. Meissner, Flávia M. Zimbres, Monika A. Coronado, Frank M. Sauer, Isolmar Schettert, Carsten Wrenger

**Affiliations:** ^1^Unit for Drug Discovery, Department of Parasitology, Institute of Biomedical Science, University of São Paulo, Avenida Professor Lineu Prestes 1374, 05508-000 São Paulo, SP, Brazil; ^2^Multi User Center for Biomolecular Innovation, Department of Physics, São Paulo State University, UNESP/IBILCE, C. Postal 136, 15054-000 São José do Rio Preto, SP, Brazil; ^3^Laboratory of Genetics and Molecular Cardiology, Heart Institute (InCor), Avenida Doctor Eneas de Carvalho Aguiar 44, 05403-000 São Paulo, SP, Brazil

## Abstract

Malaria is a deadly infectious disease which affects millions of people each year in tropical regions. There is no effective vaccine available and the treatment is based on drugs which are currently facing an emergence of drug resistance and in this sense the search for new drug targets is indispensable. It is well established that vitamin biosynthetic pathways, such as the vitamin B6 *de novo* synthesis present in *Plasmodium*, are excellent drug targets. The active form of vitamin B6, pyridoxal 5-phosphate, is, besides its antioxidative properties, a cofactor for a variety of essential enzymes present in the malaria parasite which includes the ornithine decarboxylase (ODC, synthesis of polyamines), the aspartate aminotransferase (AspAT, involved in the protein biosynthesis), and the serine hydroxymethyltransferase (SHMT, a key enzyme within the folate metabolism).

## 1. Introduction

Malaria is a devastating infectious disease, which causes serious problems in tropical and subtropical areas. According to the World Health Organization (WHO), the population of more than 100 countries is exposed to malaria parasites [1]. The causative agent of malaria is belonging to the genus *Plasmodium*, which can affect almost all vertebrates; however, only five species have been reported to be infective for humans, *P. falciparum, P. vivax, P. ovale, P. malariae, *and* P. knowlesi* [2]. The transmission of the parasite occurs via a blood meal of the *Anopheles* vector. Thereby, sporozoites are transmitted to the vertebrate host and the comprehensive life cycle of the pathogen is initiated [3]. In the past, several attempts to control the disease have been undertaken to exterminate the vector with insecticide. However, due to spreading drug resistance, these insecticides lost their efficacy [4]. A similar situation is present for the treatment of patients, since an effective vaccine is not yet available and the medication of malaria is solely based on drugs [5, 6].

The folate (vitamin B9) metabolism is a validated drug target in several infectious diseases and its biosynthesis is not present in humans. Folate is an essential cofactor in enzymatic reactions transferring one-carbon (C1) groups [7, 8] and prominent antimalarials such as pyrimethamine and cycloguanil (inhibitors of the dihydrofolate reductase) and the sulfa drugs against the dihydropteroate synthase are well characterised within the vitamin B9 metabolism [7, 8]. However—among others—resistance is also rising against this metabolic pathway. Currently, there is a move towards artemisinin-based combination therapies (ACTs) [9, 10].

As already indicated above, due to the fact that currently no effective vaccine is available and the parasite's speed in developing resistance against almost all chemotherapeutic compounds is alarming, there is an urgent need to discover novel drug-targets, which are subsequently exploitable for the design of new therapeutics against the malaria pathogen [11, 12]. In the search for novel antimalarials, attention has been drawn on selective interference with the parasite's metabolism without harming the human host [13]. In this sense promising drug targets are vitamin biosynthetic pathways.

Vitamins are molecules which have a variety of functions in nature. They act as antioxidants, as precursors in electron carrying processes, or are involved in enzymatic reactions by acting as cofactors in metabolic pathways such as the vitamins of the B-family [14]. Mammals generally depend on the uptake of vitamins, unlike other groups, such as bacteria, plants, and fungi which can synthesize them *de novo*. Some apicomplexan parasites possess also vitamin biosynthetic pathways which represent attractive drug targets to interfere with [7, 13].

**Figure 1:**
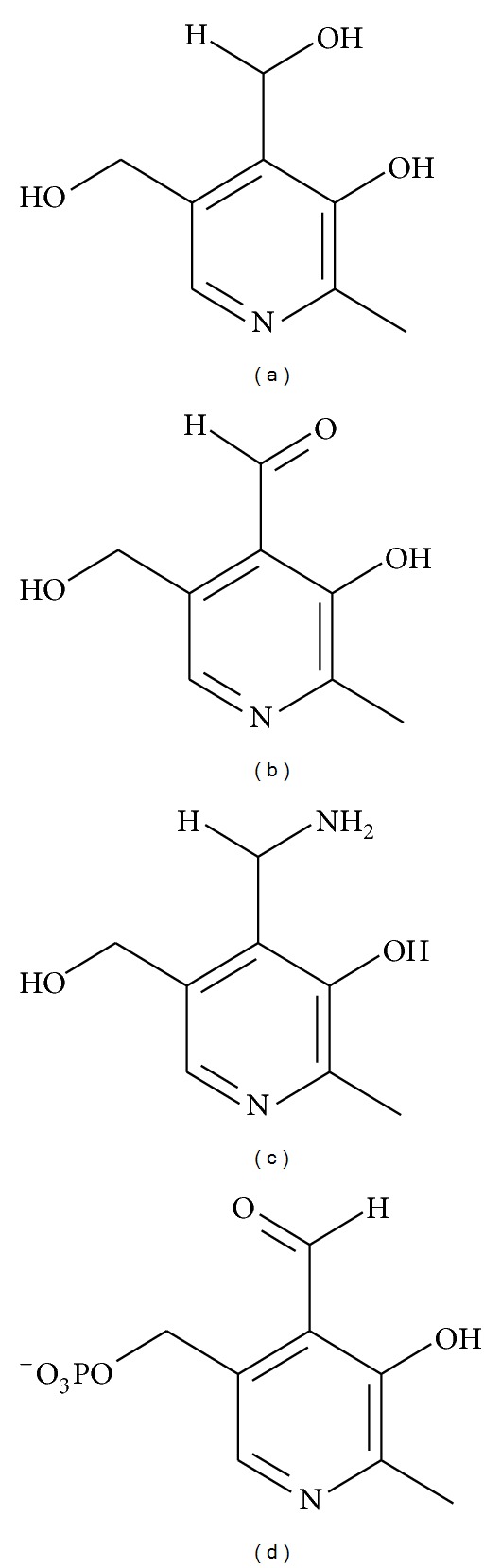
Chemical structures of vitamin B6: (a) pyridoxine, (b) pyridoxal, (c) pyridoxamine, and (d) its active form pyridoxal 5-phosphate.

So far, three vitamin biosynthetic pathways have been identified in malaria parasites [7, 13]. Besides the occurrence of the biosynthesis for folate (vitamin B9) and the thiamine (vitamin B1) biosynthesis, *Plasmodium* possesses also a vitamin B6 biosynthetic pathway. Vitamin B6 is designated for six vitamers: pyridoxine (PN), pyridoxamine (PM), pyridoxal (PL), and their respective phosphorylated forms. The differente molecules differentiate in their substitutions at the 4th position of the pyridine ring (Figure 1). However, pyridoxal 5-phosphate (PLP) is the only active form of the enzymatic cofactor which is mainly involved in decarboxylation and transamination reactions [15].

Up to now, two different vitamin B6 biosynthesis pathways are described: (i) the 1-deoxy-D-xylulose 5-phosphate (DOXP)-dependent pathway is found in some proteobacteria and is leading to pyridoxine 5-phosphate [16–18]; (ii) the second pathway, the DOXP-independent pathway, is found in plants, fungi, and the apicomplexan parasites *Plasmodium* and *Toxoplasma gondii *[19–21].

Historically, the DOXP-independent pathway was identified in plants and ascribed to oxidative stress response [22, 23]. Afterwards, the analysis of this pathway discovered the biosynthesis of PLP, which is mediated by an enzyme complex (PLP-synthase) composed of a core of 12 Pdx1 (also known as SNZ1 in yeast) individually surrounded by 12 Pdx2 (called SNO1 in yeast) [24, 25]. The reaction mechanism has already been studied in some detail, starting with the deamination of glutamine to glutamate which is catalysed by Pdx2, subsequently, the ammonia group is channelled to Pdx1, where it is combined with the two other substrates, ribose 5-phosphate and glyceraldehyde 3-phosphate, leading to the active cofactor [24, 26]. This complex has already been tested for its druggability by performing *in silico* screens in order to dock compounds into the active site. Identified compounds were further employed in *in vitro* assays using recombinantly expressed enzymes. The best compound derived from this screen was 4-phospho-D-erythronhydrazide, which revealed an IC_50_-value of 10 *μ*M in cell culture experiments [27].

Moreover, besides the well-established function of vitamin B6 in acting as a cofactor, the molecule is also involved in the combat against reactive oxygen species (ROS), in particular against singlet oxygen [22, 28]. This additional mode of action is especially of relevance for the intraerythrocytic stage of the human malaria parasite, because *Plasmodium* is permanently exposed to ROS during proliferation within the erythrocytes due to the oxidative environment of its host cell which is accompanied by the parasite-driven haemoglobin degradation [29, 30].

Additionally, the parasite's genome encodes also for an interconversion pathway which consists of the pyridoxal kinase (PdxK) and a phosphatase [4, 7]. The latter reveals a broad substrate spectrum and therefore it is questionable whether this enzyme is solely responsible for the dephosphorylation of B6 vitamers [20, 31]. The PdxK catalyses the phosphorylation of pyridoxal but also accepts the other B6 vitamers as substrate [20, 32]. The presence of both—biosynthetic and interconversion—pathways remains still for elucidation since the parasite is able to generate PLP via two pathways which would obviously emphasise an uptake of B6 vitamers [4].

In *P. falciparum,* the PdxK enzyme was already exploited as drug target by channelling prodrugs into the parasite's metabolism. Pyridoxyl-tryptophan chimeras were converted into their respective phosphorylated forms by the PdxK. Subsequently, these molecules were shown to interfere with PLP-dependent enzymes by inhibiting their catalyses and hence the growth of the parasite [32].

## 2. PLP-Dependent Enzymes

PLP-dependent enzymes are characterised by their broad range of enzymatic activities and their participations in different metabolic pathways [15, 52]. They are mainly concentrated within the amino-acid metabolism [53]. Besides the glycogen phosphorylases, which follow a different mechanism [54, 55], PLP-dependent enzymes bind PLP during catalysis covalently to the respective substrate by acting as an electrophilic stabilizer of the carbanion intermediate [56]. In the past, a few attempts have been undertaken to classify PLP-dependent enzymes according to their activities and evolutionary history by splitting them into four major classes [57, 58]. Due to their conservation in the nature, it has been suggested that PLP-dependent enzymes derived from a common ancestor before division into the three kingdoms of life occurred [57].

Afterwards, this classification was refined by analysing genomic and structural information [15, 33] which led to the sorting of PLP-dependent enzymes into seven groups (Table 1).

Kappes and collaborators suggested that, because of the existing metabolic diversity, PLP-dependent enzymes in protozoan parasites would have potential to be good drug targets [59]. Most of the enzymes found (at least 2/3) belong to group I, followed by the less expressive group II, while the groups IV and V are rare and the groups VI and VII are almost inexistent. Recent genome database analyses of different parasites identified a minimal set of enzymes that are highly abundant which includes the serine hydroxymethyltransferase (SHMT), the aspartate aminotransferase (AspAT), the alanine transaminase, the branched-chain amino-acid transaminase, and the cysteine desulfurase [59].

Moreover, the comparison of all available genomes of free-living organisms revealed that only two EC-classified enzymes are always present: the AspAT (EC 2.6.1.1) and the SHMT (EC 2.1.2.1), which underlines the fundamental importance of these enzymes [15].

Additionally, several other PLP-dependent enzymes have already been exploited as drug targets such as the *γ*-aminobutyric acid GABA aminotransferase by the drug vigabatrin for treatment of epilepsy [60], the alanine racemase in microbicides [61], or the ornithine decarboxylase (ODC) in cancer research [62]. In particular, the ODC was also subject to drug discovery approaches against protozoan parasites but not limited as outlined below to the aspartate aminotransferase (AspAT) and the serine hydroxymethyltransferase (SHMT). However, the occurrence of PLP-dependent enzymes in the malaria parasite is not restricted to these three proteins as shown in Table 2.

## 3. Ornithine Decarboxylase (ODC)

As already outlined above, vitamin B6-dependent enzymes play central roles not only in the metabolism of amino acids but also in the polyamine synthesis. Polyamines are simply structured aliphatic nitrogenous bases containing an essential role in cell growth, proliferation, and differentiation due to their stabilizing effect on macromolecules such as nucleic acids, proteins, and lipids. Their function is considered to be based on reversible ionic interactions with the negatively charged macromolecules [63–65].

**Figure 2:**
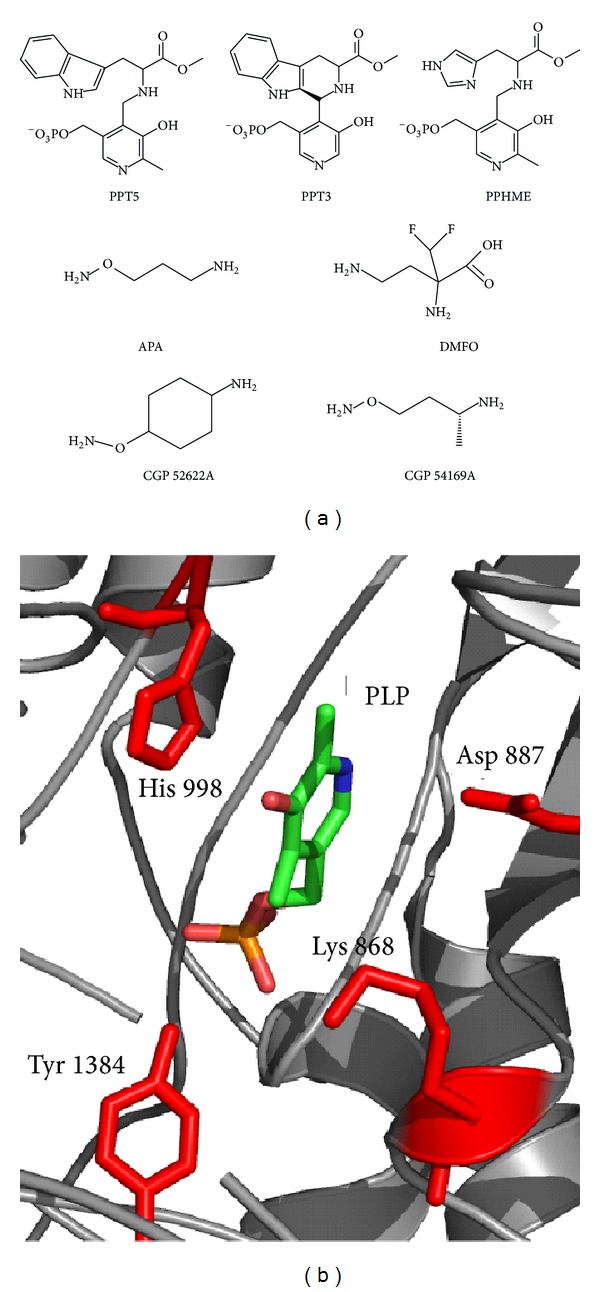
Comparison of the active site of the human and plasmodial ornithine decarboxylases (ODC). (a) Structures of ODC inhibitors tested against *Plasmodium*. (b) A structural homology model of the positions of the *P. falciparum* ODC active site (the respective residues are illustrated in red; amino acid numbering refers to the bifunctional protein) as well as the bound cofactor PLP.

The ornithine decarboxylase (ODC) is a PLP-dependent enzyme (Figure 2) which acts as a key regulator in the polyamine biosynthesis by decarboxylating ornithine to the polyamine putrescine—the first step in this synthesis. In contrast to ornithine, the other precursor of the polyamine synthesis, *S*-adenosylmethionine (AdoMet), is synthesized from methionine and ATP by the enzyme AdoMet synthase. AdoMet is also used to generate the polyamines spermidine and spermine. *P. falciparum* possesses a unique polyamine biosynthesis due to the bifunctional organisation of its key enzymes, *S*-adenosylmethionine decarboxylase (AdoMetDC) and ornithine decarboxylase (ODC) [42, 66]. Thereby, both enzymes appear as the bifunctional AdoMetDC/ODC whose organisation was discussed as an advantage in substrate channelling [66].

There are more bifunctional proteins known in *P. falciparum* such as the dihydrofolate reductase-thymidylate synthase (DHFR-TS) which is also present in other protozoa [67, 68], the dihydro-6-hydroxymethylpterin pyrophosphokinase-dihydropteroate synthase (PPPK-DHPS) [69], the glucose-6-phosphate dehydrogenase/6-phosphogluconolactonase [70], and the guanylate cyclase/adenylate cyclase [71].

Among others, this unique organisation of the *Pf*ODC has been discussed to be an attractive drug target [72]. As the amino acid sequence of *Pf*ODC shares about 39% identity to the human homologue, complications in rational drug design of *Pf*ODC-specific lead compounds could be a crucial issue [39]. Generally, there are three different strategies of inhibitor design. A formerly used strategy for designing inhibitors of vitamin B6-dependent enzymes is based on coenzyme-substrate conjugates that cannot be processed by the enzyme in their reduced form [73].

Another—already validated—strategy is the use of substrate analogues in order to inhibit enzyme catalysis like the specific ODC inhibitor difluoromethylornithine (DFMO), originally designed as an anticancer agent. DFMO blocks the erythrocytic schizogony of *P. falciparum* in cell culture at the micromolar level (Table 2) and reduces the parasitemia in *Plasmodium berghei*-infected mice [47, 48, 74, 75]. DFMO, a derivative of ornithine, inhibits the enzyme irreversibly by an alkylation of its active site. A combination of DFMO and bis(benzyl)polyamines revealed a curative effect in rodent malaria [76]. Moreover, DFMO reveals a more prominent role due to its effectiveness against *Trypanosoma brucei gambiense*, the agent of the West African Sleeping Sickness [77–79]. Only marginal effects of DFMO have been observed against the apicomplexan relatives of *P. falciparum*, *Cryptosporidium sp*. [80] and *Toxoplasma gondii *[50].

Furthermore, two decades ago, a series of potent ODC inhibitors were synthesized. These compounds belong to the group of 3-amino-oxy-1-propanamine (APA) [81, 82], such as CGP52622A and CGP54619A (Figure 2), which reversibly inhibit the *Pf*ODC with IC_50_-values at the nanomolar range (Table 3). APA itself had an IC_50_-value of 1 *μ*M revealing a 1000-fold stronger antiplasmodial effect than DFMO (IC_50_ value of 1.3 mM) (Table 3). However, APA and its analogues failed as drug candidates in the mouse model [83].

Another interesting PLP-mimicking compound is the cyclic pyridoxyl-tryptophan methyl ester PT3 which inhibits in its phosphorylated form (PPT3) the proliferation of *P. falciparum* at the cellular level (IC_50_-value of 14 *μ*M) without harming human cells [32]. Two further compounds of this chemical group, PPHME and PPT5, act as inhibitors of the plasmodial ODC with IC_50_-values of 58 *μ*M and 64 *μ*M, respectively [32] (Figure 2).

## 4. The *P. falciparum* Aspartate Aminotransferase (AspAT)

Aspartate aminotransferases are involved in three different metabolic pathways. AspAT is responsible for the reversible catalysis of L-aspartate (Asp) into oxaloacetate (OAA) and *α*-ketoglutarate (2OG) into L-glutamate (Glu) [37]. Bulusu and collaborators [84] highlighted that AspAT also acts together with the fumarate hydratase (FH) and the malate-quinone oxidoreductase (MQO) in the conversion of fumarate to aspartate. The enzyme has also been described to accept *α*-ketomethylthiobutyrate as substrate in order to generate methionine [85]. Like all other aminotransferases, AspAT is structurally classified as a PLP-dependent enzyme of the subgroup I as outlined previously (Table 1) [86].

**Figure 3:**
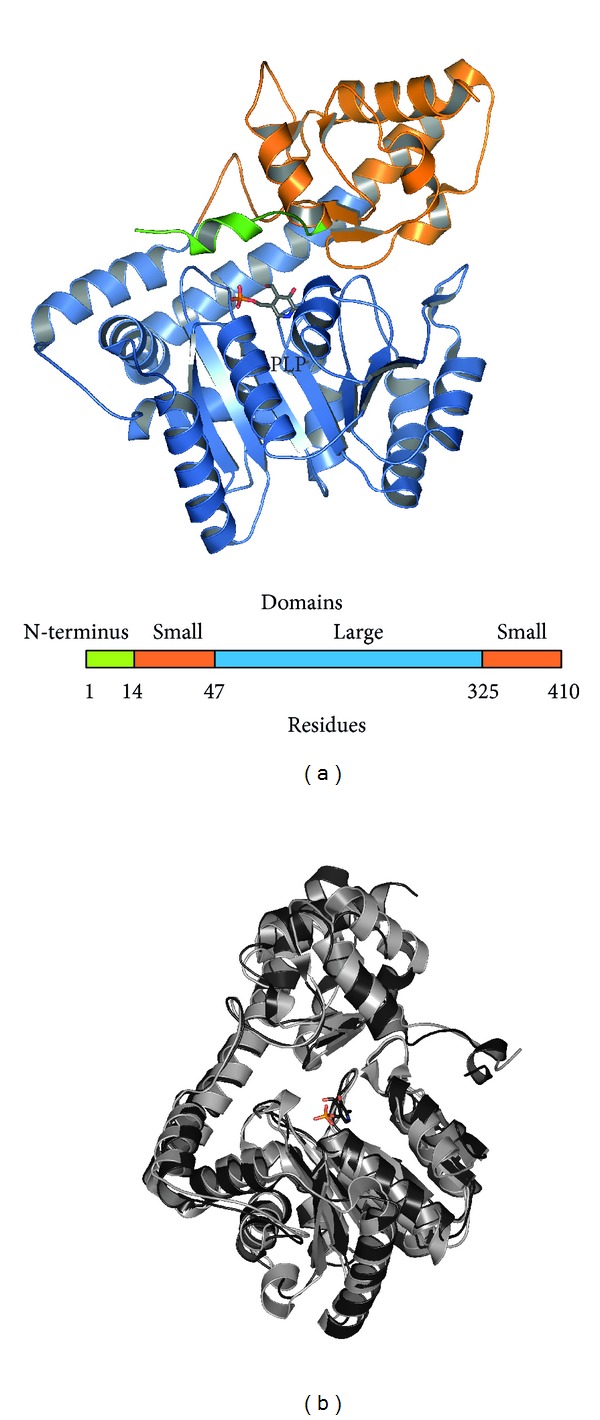
Three-dimensional structures of the AspATs. (a) The 3D structure of *Pf*AspAT (PDB code: 3K7Y) highlighting the three major domains and the N-terminus (green) as additionally shown in the scheme below. (b) Comparison between the human AspAT (grey, PDB code: 3HLM) and the *P. falciparum* counterpart (dark grey). The respective N-terminal region is illustrated in black and the cofactor PLP in colour.

In the malaria parasite, AspAT is localised in the cytosol and reveals a homodimeric structure with two joint active site regions formed by both subunits [87–89]. Special attention has been drawn on the plasmodial AspAT (PDB code 3K7Y) which possesses a N-terminal-extended region that is required for the dimerisation process (Figure 3) [37]. This was already used for binding of the N-terminal AspAT peptide to the N-terminal protein domain of the other *Pf*AspAT monomer which prevents the formation of the homodimer. Interestingly, the plasmodial N-terminal region differs significantly from its human counterpart, so that the plasmodial peptide did not affect the human AspAT [37]. Furthermore, activity assays using *P. falciparum* protein extracts and the recombinantly expressed N-terminal *Pf*AspAT peptide have been performed which prevented AspAT activity suggesting that the malaria parasite possesses no other enzyme that can compensate for the respective catalysis [37, 89].

## 5. Serine Hydroxymethyltransferase (SHMT)

As mentioned before, the folate metabolism in *P. falciparum* is a verified drug target and enzymatic reactions catalysed, for example, by the dihydrofolate reductase (DHFR) are already exploited by the classic antimalarials pyrimethamine and cycloguanil [90]. Another enzymatic step within the folate metabolism is carried out by the serine hydroxymethyltransferase (SHMT), catalysing the transfer of one-carbon units from serine to tetrahydrofolate to generate 5,10-methylene tetrahydrofolate and glycine; this *α*-elimination catalysis is PLP-dependent, thereby belonging to the subgroup I [91].

The folate metabolism is of particular interest because it is involved in the pyrimidine biosynthesis which is required for the DNA synthesis. Since the SHMT is part of the folate metabolism, its transcription profile is increased in the *S*-phase of the DNA replication [92]. Due to the importance of this enzyme, SHMT is considered as a potential drug target in cancer research [93, 94]. In this sense, inhibitors against tumour cells have already been developed, which are intended to mimic nucleosides in order to be subsequently incorporated into the DNA, thereby leading to its fragmentation [95]. The SHMT of *P. falciparum* has been analysed for its functionality by complementation assays in *Escherichia coli* [96]. Moreover, activity assays using the recombinantly expressed *Pf*SHMT showed that the enzyme accepts in addition to the natural substrate—unlike its mammal counterpart—D-serine. This lack of stereospecificity has also been observed for the respective *P. vivax* enzyme [97]. Further, the plasmodial enzyme can be also inhibited competitively by glycine and serine [34].

**Figure 4:**
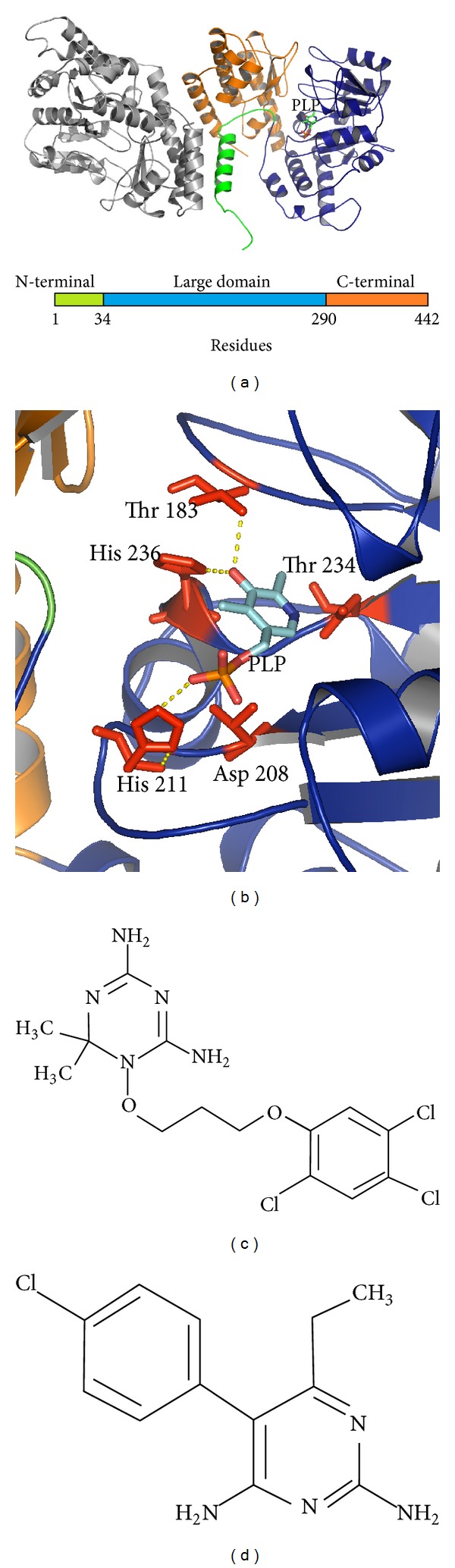
Model of the plasmodial SHMT and their active site residues. (a) Homology model of the SHMT of *P. falciparum *highlighting the three major domains: N-terminal (green), the core and active site (blue), and the C-terminal domain (orange). (b) The conserved residues Asp208, His211, Thr234, His236 and the *Plasmodium*-specific Thr183 residue are illustrated within the active site of the *Pf*SHMT as well as its embedded cofactor. Chemical structures of validated inhibitors of the folate metabolism (c) WR99210 and (d) pyrimethamine.

Since the substrates of SHMT and DHFR are structurally similar (Figure 4), pyrimethamine, a potent inhibitor of the plasmodial DHFR, has also been tested on the recombinant SHMT, however, only with a marginal effect (IC_50_-value in the midmicromolar range) [35]. The comparison between the active site of the human enzyme and the plasmodial one showed a high degree of similarity as illustrated in Figure 4 [98], but, in contrast to the mammalian SHMT, which reveals a homotetrameric structure, the structural conformation of the plasmodial protein pointed towards a homodimeric appearance due to the lack of amino acid residues proposed to be involved in tetramerisation (like the His 135 and a poly-K sequence within the N-terminal domain) [98].

Despite all the similarities between the human and the malaria SHMT, the plasmodial enzyme possesses some peculiarities in the regulation of the folate metabolism such as binding to its own RNA [35], thus inhibiting protein translation [99].

Recently, a second open reading frame encoding for a potential mitochondrial SHMT (PF14_0534, mSHMT) has been identified in *P. falciparum.* However, in comparison to other SHMTs, the active site of the plasmodial mSHMT does not reveal preserved amino acid residues [35, 100].

## 6. Druggable PLP-Dependent Enzymes in the Malaria Vector

Within the life cycle of *P. falciparum,* the necessity of PLP-dependent enzymes is not only restricted to the parasite. In order to complete its life cycle, sexual forms of the parasite have to be taken up via the blood meal of the *Anopheles* vector to enter the mosquito gut [3]. Subsequently, the gametogenesis is induced in the mosquito stage by *Anopheles *derived triggers [101]. One of these molecules, that has been described to play a role in this event, is xanthurenic acid (XA) [101]. XA is generated by a transamination reaction of 3-hydroxykynurenine (3-HK) which is catalysed by the PLP-dependent *A. gambiae* 3-HK transaminase (*Ag*HKT), an enzyme classified to the subgroup I. This reaction is necessary to prevent accumulation of the 3-HK, which can become a toxic molecule if it undergoes spontaneous oxidation and thereby generates ROS [45, 101]. The three-dimensional structure of the recombinant *Ag*HKT was solved as a homodimer with a PLP molecule located in its active site [58, 86]. Currently, there are no inhibitors known to target the *Ag*HKT, although structural information would enable *in silico *based drug-design [45]. Selective interference with the mosquito HKT would prevent the synthesis of XA and thereby offers the opportunity to block the life cycle of the malaria parasite in the mosquito stage.

## 7. Conclusion

Although the mortality of malaria infections is declining, the disease, of which malaria tropica (caused by *P. falciparum*) is the most fatal form, belongs still to the most important infectious disease to man. Due to the increasing level of resistance against the current chemotherapeutics, there is an urgent need to discover novel drugs which should have the ability to selectively interfere with the proliferation of this human pathogen. In this sense, the unique plasmodial cofactor metabolism becomes an attractive drug target due to the variety of cofactor-dependent enzymes. In particular, PLP-dependent enzymes are widely distributed in the metabolism of *P. falciparum* and responsible for plenty of essential catalyses such as the reactions carried out by the ODC, AspAT, or SHMT as outlined in this minireview. Hence, drug discovery towards inhibition of cofactor-binding would not only target single enzymes; moreover, the entire family of PLP-dependent proteins would be affected. This would certainly lead to the death of the parasite. However, the respective PLP-dependent host enzymes have to be taken into account. Therefore, the selective impairment of the malaria specific vitamin B6 biosynthesis should be considered.

## Figures and Tables

**Table 1:** Different classes of PLP-dependent enzymes according to [15, 33].

Group number	Enzyme class/activity	Representative enzymes
1	Aminotransferases and the amino-acid decarboxylases	Serine hydroxymethyltransferase (SHMT) and the aspartate aminotransferase (AspAT, prototype)
2	Replacement and elimination of C_*β*_-groups	Serine and threonine dehydratases and the tryptophan synthase (prototype)
3	Interconversion of L- and D-amino acids with a common folding (alpha/beta)_8_	Alanine racemase
4	Alanine aminotransferase	D-Alanine aminotransferase
5	Glycogen phosphorylase	Glycogen phosphorylase
6	5,6-Aminomutase	D-Lysine 5,6-aminomutase
7	2,3-Aminomutase	Lysine 2,3-aminomutase

**Table 2:** PLP-dependent enzymes in *Plasmodium*.

EC-number	EC-name	PlasmoDB number	Annotation according to PlasmoDB	Pathway	Inhibitors	References
2.1.2.1	Glycine hydroxymethyltransferase	PFL1720w	Serine hydroxymethyltransferase	Folate metabolism	1843U89, AG331, AG337, D1694, GR1, pemetrexed, pyrimethamine, WR99210, methotrexate, glycine (competitively)	[34, 35]
2.3.1.37	5-Aminolevulinate synthase	PFL2210w	ALA synthase (aminolevulinate synthase)	Tetrapyrrole biosynthesis	Aminomalonate, Ethanolamine, Hemin	[36]
2.6.1.1	Aspartate aminotransferase	PFB0200c	Aspartate aminotransferase	Amino acid and pyrimidine metabolism	Inhibited by his own N-terminal peptide	[37]
2.6.1.13	Ornithine aminotransferase	PFF0435w	Ornithine aminotransferase	Argnine metabolism	L-canaline	[38]
2.6.1.57	Aromatic amino-acid transaminase	PFB0200c	Aspartate aminotransferase	Amino acid and pyrimidine metabolism	—	—
4.1.1.17	Ornithine decarboxylase	PF10_0322	*S*-Adenosylmethionine decarboxylase/ornithine decarboxylase (bifunctional)	Polyamine biosynthesis	Alpha-difluoromethylornithine, alpha-difluoroornithine, CGP52622A, CGP54619A, putrescine (feedback control)	[39–44]
4.1.3.38	*p*-Aminobenzoic acid synthetase	PFI1100w	*p*-Aminobenzoic acid synthetase, putative	Folate biosynthesis	—	—
2.6.1.7	3-Hydroxykynurenine transaminase		Present in the insect vector: *Anopheles *	Xanthurenic acid is needed by the parasite for proliferation/ development	—	[45]

Putative PLP-dependent enzymes						
2.3.1.50	Serine C-Palmitoyltransferase	PF14_0155	Serine C-Palmitoyltransferase	Sphingolipid metabolism	—	—
2.6.1.42	Branched-chain amino-acid aminotransferase	PF14_0557	“Conserved *Plasmodium* protein”	Pantothenate and CoA biosynthesis	—	—
2.8.1.7	Cysteine desulfurase	PF07_0068, MAL7P1.150	Cysteine desulfurase, putative	Iron-sulfur cluster synthesis	—	—
4.1.1.18	Lysine decarboxylase	PFD0285c, PFD0670c	Lysine decarboxylase, putative	Polyamine metabolism	—	—

**Table 3:** Comparison of the kinetic and inhibitory properties of ornithine decarboxylases.

	P. falciparum*	*M. musculus *	*T. gondii *	*T. brucei *	References
Molecular mass (kDa)	86.4	50–54	14	90	[41, 46]
*K*_*m*_-value of L-ornithine (*μ*M)	47.3	30–200	—	161	[41, 47, 48]
*K*_*i*_-value of putrescine (*μ*M)	50.4	600	0.92	—	[41, 49]
*K*_*i*_-value of DFMO (*μ*M)	87.6	39	0.025	220	[41, 50, 51]
*K*_*i*_-value of CGP52622A (nM)	20.4	—	—	—	[41]
*K*_*i*_-value of CGP54619A (nM)	7.9	—	—	—	[41]
IC_50_-value of putrescine (*μ*M)	157	—	—	—	[41]
IC_50_-value of CGP52622A (nM)	63.5	25	—	—	[41]
IC_50_-value of CGP54619A (nM)	25	10	—	—	[41]

*Data derived from the r*Pf* hinge-ODC [41].
